# Evolution of size‐dependent intraspecific competition predicts body size scaling of metabolic rate

**DOI:** 10.1111/1365-2435.13253

**Published:** 2018-12-20

**Authors:** Vincent Hin, André M. de Roos

**Affiliations:** ^1^ Institute for Biodiversity and Ecosystem Dynamics University of Amsterdam Amsterdam The Netherlands

**Keywords:** asymmetric intraspecific competition, body size, dynamic energy budgets, metabolic scaling

## Abstract

1. Growth in body size is accompanied by changes in foraging capacity and metabolic costs, which lead to changes in competitive ability during ontogeny. The resulting size‐dependent competitive asymmetry influences population dynamics and community structure, but it is not clear whether natural selection leads to asymmetry in intraspecific competition.
2. We address this question by using a size‐structured consumer–resource model, in which the strength and direction of competitive asymmetry between different consumer individuals depends on the scaling of maximum ingestion and maintenance metabolism with consumer body size. We use adaptive dynamics to study selection on the scaling exponents of these processes.
3. Selection leads to an identical scaling of maximum ingestion and maintenance metabolism with consumer body size. Equal scaling exponents neutralize strong competitive differences within the consumer population, because all consumer individuals require the same amount of resources to cover maintenance requirements. Furthermore, the scaling exponents respond adaptively to changes in mortality such that biomass production through growth or reproduction increases in the life stage that is subject to increased mortality. Also, decreasing size at birth leads to increased investment in juvenile growth, while increasing maximum size leads to increased investment in post‐maturation growth and reproduction.
4. These results provide an explanation for observed variation in the ontogenetic scaling of metabolic rate with body size. Data of teleost fish are presented that support these predictions. However, selection towards equal scaling exponents is contradicted by empirical findings, which suggests that additional ecological complexity beyond this basic consumer–resource interaction is required to understand the evolution of size‐dependent asymmetry in intraspecific competition.

A plain language summary is available for this article.

## 1 INTRODUCTION

Intraspecific competition is often asymmetric such that some members of a population have a large negative effect on others, but suffer little from competition themselves. Furthermore, this asymmetry often depends on individual body size. For example, small larvae of the damselfly *Ischnuru elegans* suffer from reduced growth and longer development times due to interference from large larvae, but not from other small larvae (Gribbin & Thompson, 1990). In case of resource (or exploitative) competition, competitive ability of individuals increases with energy assimilation rate and decreases with metabolic maintenance costs (Persson, Leonardsson, De Roos, Gyllenberg, & Christensen, 1998; Werner, 1994). Energy assimilation has various behavioural and physiological components, such as attack rate, handling time and assimilation efficiency, which can scale with body size in different ways (Persson et al., 1998; Peters, 1983). The nature of the scaling relationships of energy assimilation and metabolic maintenance costs with body size ultimately determine whether large or small individuals are competitively superior and therefore able to grow or reproduce with the available resources. In fish, for example, metabolic costs generally increase faster with body size than energy assimilation rates (Persson, 1985; Persson & De Roos, 2006), which means that larger individuals require more resources to cover maintenance requirements. This can lead to starvation of large individuals when many small conspecifics suppress resource density. These consequences of asymmetric intraspecific competition are often found in freshwater fish species that predominantly feed on the same resource (Edeline, Terao, & Naruse, 2016; Hjelm & Persson, 2001; Persson, 1985; Sanderson, Hrabik, Magnuson, & Post, 1999; Ward, Webster, & Hart, 2006).

Size‐dependent asymmetry in intraspecific competition has large consequences for population dynamics, species coexistence and community structure (De Roos & Persson, 2013; De Roos, Persson, & McCauley, 2003). Concerning population dynamics, the occurrence and type of population cycles has been linked to the body‐size scaling of competitive ability, as measured by the maintenance resource density (MRD; Persson et al., 1998). Sometimes referred to as critical resource density (Byström & Andersson, 2005; Persson & De Roos, 2006), the MRD is the resource density that an individual requires to cover its maintenance metabolism, with superior competitors having a lower MRD. An increase in the MRD with body size leads to population cycles induced by competitive superiority of small individuals (juvenile‐driven cycles: De Roos & Persson, 2013), while a decrease of the MRD with body size implies that large individuals are competitively superior and this causes adult‐driven cycles.

Consequences of size‐dependent competitive asymmetry for species coexistence and community structure are mediated through changes in the population size distribution. When competitive ability changes with body size, either large or small individuals produce more biomass per unit of existing biomass (i.e., they have a higher mass‐specific biomass production rate). Generally, newly produced biomass is allocated to reproduction by adults and to somatic growth by juveniles. Stage‐specific differences in mass‐specific biomass production lead to differences in rates of biomass transfer between these different life stages, with biomass maturation rates being higher (lower) than biomass reproduction rates when juveniles (adults) have a higher mass‐specific biomass production rate. This creates an energetic bottleneck in the flow of biomass across the life cycle as biomass accumulates in the life stage with the lowest mass‐specific production rate (De Roos et al., 2007). Increasing (size‐ or stage‐specific) mortality alleviates such a bottleneck and causes an overcompensatory increase of biomass in the other life stage (De Roos et al., 2007). This phenomenon of biomass overcompensation is shown to occur in both experimental (Cameron & Benton, 2004; Schröder, Persson, & De Roos, 2009) and natural systems (Ohlberger et al., 2011) and can lead to community wide effects such as emergent Allee effects (De Roos, Persson, & Thieme, 2003), emergent facilitation between two size‐selective predators (De Roos, Schellekens, Van Kooten, & Persson, 2008) and alternative stable states (Guill, 2009).

Besides its impact on population dynamics and community structure, asymmetric competition can considerably influence individual life history, with potential evolutionary consequences. A modelling study based on Trinidadian guppies revealed that the degree of asymmetry in competition changed both mean and variance of generation time and life expectancy at birth and also the variance of lifetime reproductive success (Bassar et al., 2016). This study suggests that asymmetric competition can influence the direction and speed of evolutionary life‐history changes through changes in the nature of density dependence, although explicit predictions were not discussed. Evolutionary consequences of intraspecific competition have mainly been studied in the light of ecological character displacement, where increased competition leads to diversification in diet and morphology between individuals (Svanbäck & Bolnick, 2007). It remains unclear how the degree and direction of asymmetry in competition affects eco‐evolutionary dynamics and under which conditions natural selection would lead to symmetry or asymmetry in intraspecific competition.

The scaling of energy assimilation and maintenance metabolism with body mass is often allometric and can be described by a power function that contains a proportionality constant and a scaling exponent (Glazier, 2005; Peters, 1983). Two frameworks provide a value for the scaling exponents of assimilation and maintenance metabolism: the ontogenetic growth model of West, Brown and Enquist (OGM‐model; Hou, Zuo, Moses, & Woodruff, 2008; West, Brown, & Enquist, 2001) and dynamic energy budget (DEB) theory (Kooijman, 2010). In both frameworks, ontogenetic growth results from the difference between resource or energy supply and maintenance costs of existing cells or structure (Kearney & White, 2012; Maino, Kearney, Nisbet, & Kooijman, 2014; Van der Meer, 2006). In the OGM‐model energy supply is proportional to the resting metabolic rate, which is assumed to scale with three‐quarters power of body mass (Hou et al., 2008; West et al., 2001). This three‐quarters scaling follows from an independent model of a distribution network that delivers resources to terminal units (capillaries). Minimization of the energetic costs in such a network leads to a fractal‐like distribution network in which the number of terminal units scales with three‐quarters power to body mass (West, 1997; West, Brown, & Enquist, 1999). DEB theory describes an individual in terms of structural body volume and reserve density. Resource supply is assumed proportional to structural surface area and therefore scales with a two‐thirds power of structural volume for isomorphically growing organisms, while maintenance costs increase isometrically with volume (Kooijman, 1986, 2010).

Recently, data are accumulating that indicate substantial variation in the value of the scaling exponent of metabolic rate and this variation has been related to taxonomic diversity, lifestyle in aquatic organisms (pelagic vs. non‐pelagic), temperature, life stage, activity level, physiological state, predation and body shape (Glazier, 2005, 2006, 2009; Glazier, Hirst, & Atkinson, 2015; Glazier et al., 2011; Hirst, Glazier, & Atkinson, 2014; Killen, Atkinson, & Glazier, 2010). Glazier (2005) argues that the diverse scaling relationships observed in nature result from diverse adaptations in combination with ecological physico‐chemical constraints. This suggests that scaling exponents can change adaptively, for example, through changes in body shape during ontogeny (Hirst et al., 2014; Killen et al., 2010; Okie, 2013). Such changes would alter the degree of competitive asymmetry within the population and have substantial consequences for population and community dynamics, as well as individual life history. However, explicit predictions about the evolutionary dynamics of competitive asymmetry are lacking. Therefore, the main purpose of this study is to understand the selection pressures that act on the scaling of competitive ability with body size, as determined by the body‐size scalings of energy assimilation and maintenance metabolism.

To this aim, we formulate a size‐structured consumer–resource model in which ingestion, maintenance, growth and reproduction of consumer individuals are described by a DEB model. Energy assimilation is proportional to resource ingestion, and both maximum ingestion and maintenance rates follow power functions of body mass. The individual‐level DEB model is translated to the population level by considering the density distribution of individuals along the body mass axis, as in the framework of physiologically structured population models (Metz & Diekmann, 1986). We explore how the scaling exponents of maximum ingestion and maintenance affect population dynamics and subsequently study the evolutionary dynamics that result from selection pressures on these scaling exponents. Fitness and the direction and strength of selection arise through the feedback between an individual and its environment. Therefore, we use the framework of adaptive dynamics (Geritz, Kisdi, Meszena, & Metz, 1998; Metz, 2012) to study evolutionary change and identify evolutionary endpoints of the scaling exponents of ingestion and maintenance rates.

## 2 MODEL DESCRIPTION

### 2.1 DEB model

The DEB model (Table 1) specifies rates of resource ingestion, maintenance, growth, reproduction and mortality of a consumer individual, as a function of its body mass *s* and resource density *R*. Resource competition between individuals is explicitly incorporated by considering a self‐replenishing, dynamic resource from which all consumer individuals feed and by modelling growth and reproduction as food‐dependent processes. Allocation of assimilated energy from resource feeding follows a net‐production allocation scheme, in which maintenance takes precedence over growth and reproduction (Lika & Nisbet, 2000). Therefore, growth and reproduction only occur when resource density is more than sufficient to cover maintenance requirements.

**Table 1:** Model equations

Equation	Description
$I\left(R,s\right)\;=\;M{\left(\frac{s}{s_{r}}\right)}^{Q}\frac{R}{R\;+\;H}$	Resource ingestion
$\Omega \left(R,\;s\right)\;=\;\sigma I\left(R,s\right)\;-\;T{\left(\frac{s}{s_{r}}\right)}^{P}$	Biomass production
$\frac{\text{d}s(R,\;a)}{\text{d}a}\;=\;g\left(R,\;s\right)\;=\;\kappa \left(s\right)\Omega^{+}\left(R,\;s\right)$ with $s\left(R,\;0\right)\;=\;s_{b}$	Growth rate
$b\left(R,\;s\right)\;=\;\frac{\left(1-\kappa \left(s\right)\right)\Omega^{+}\left(R,\;s\right)}{s_{b}}$	Fecundity rate
$\kappa \left(s\right)\;=\;\left\{\begin{array}{cc} 1 & fors\;<\;s_{j}\\ 1\;-\;3L{\left(s\right)}^{2}\;+\;2L{\left(s\right)}^{3} & fors_{j}\leq s\;<\;s_{m}\\ 0 & fors\;=\;s_{m} \end{array}\right.$ with $L\left(s\right)\;=\;\frac{s\;-\;s_{j}}{s_{m}\;-\;s_{j}}$	Allocation function
$\mu \left(R,\;s\right)\;=\;\left\{\begin{array}{cc} \mu_{c}\;+\;\mu_{j}\;-\;\frac{\Omega^{-}\left(R,\;s\right)}{s} & fors\;<\;s_{j}\\ \mu_{c}\;+\;\mu_{a}\;-\;\frac{\Omega^{-}\left(R,\;s\right)}{s} & fors\geq s_{j} \end{array}\right.$	Mortality rate
$G\left(R\right)\;=\;\delta \left(R_{\max}\;-\;R\right)$	Resource growth rate

*Note*

We use Ω+(R,s) to denote max(Ω(R,s),0) and $\Omega^{-}\left(R,\;s\right)$ means min(Ω(R,s),0).

Maintenance and maximum ingestion rates follow power functions of body mass, given by $T{\left(s/s_{r}\right)}^{P}$ and $M{\left(s/s_{r}\right)}^{Q}$, respectively. In these functions, body mass *s* is divided by parameter $s_{r}$. This parameter acts as the rotation point for the power functions of maintenance and maximum ingestion and is referred to as the reference body mass. The scaling constants *T* and *M*, respectively, indicate the maintenance and maximum ingestion rate of an individual with reference body mass $s_{r}$. The exponents *P* and *Q* determine the body mass scalings. An increase in *P* and *Q* implies an increase in, respectively, maintenance and maximum ingestion for individuals with *s* > *s*_r_ and a decrease for individuals with *s* < *s*_r_. Individuals are born with size $s_{b}$, mature at size $s_{j}$ and can reach a maximum size of $s_{m}$, if food density is sufficient. For $s_{b}\;<\;s_{r}\;<\;s_{m}$, there is a trade‐off between individuals with $s\;<\;s_{r}$ and conspecifics with *s* > *s*_r_, for both maintenance and maximum ingestion rates. By default a juvenile–adult trade‐off is assumed by setting $s_{r}\;=\;s_{j}$, but deviations from this assumption are explored. The rate of food ingestion (*I*(*R*,* s*)) furthermore follows a Holling type‐II functional response of resource biomass with half‐saturation constant *H* (see Table 1). Ingested food is assimilated with efficiency *σ*, which also includes overhead costs for somatic growth and reproduction. The biomass production rate (Ω(R,s)) is the amount of biomass that can be used for growth and reproduction per unit of time and is equal to assimilated biomass minus maintenance costs (Table 1).

To prevent individuals from growing to very large body sizes in case maximum ingestion increases faster with body size than maintenance (i.e., Q>P), we model adult allocation towards growth with a sigmoid function κ(s) that decreases from one at maturation size $s_{j}$ towards zero at the maximum individual body size $s_{m}$ (Table 1). Consequently, asymptotic size is limited to $s_{m}$ if resource density is sufficient, but this size might not be reached if resources are limited. In adults, the fraction of biomass production not spend on growth (1-κ(s)) is allocated to reproduction. Therefore, adult individuals allocate an increasing fraction of their biomass production towards reproduction. Juveniles ($s_{b}\leq s\;<\;s_{j}$) spend all biomass production on growth. Growth in mass with age *a* occurs only if the biomass production rate, Ω(R,s), is positive and is given by the differential equation $\frac{\text{d}s(R,\;a)}{\text{d}a}$ in Table 1. The size at birth $s_{b}$ is used as initial condition of this differential equation. Reproduction also only occurs for positive values of Ω(R,s) and individual fecundity is denoted by b(R,s) (rate of offspring production per adult; Table 1).

Mortality (μ(R,s)) is composed of background mortality for all individuals ($\mu_{c}$) and additional size‐dependent mortality for juveniles ($\mu_{j}$) and adults ($\mu_{a}$; Table 1). Furthermore, mortality increases when food conditions are insufficient to cover maintenance requirements. This starvation mortality is equal to the magnitude of the mass‐specific biomass production when negative. Starvation is handled as an increase in mortality instead of a reduction in body mass (De Roos et al., 2007). These particular starvation dynamics will not influence the evolutionary predictions of the model, since these assume the population to be at equilibrium, under which starvation does not occur. Resource growth (G(R)) follows semi‐chemostat dynamics (Table 1).

Model parameters and their default values are summarized in Table 2 and a more detailed description of the parameter derivation is available in Supporting Information Appendix S1 (see also De Roos & Persson, 2013).

**Table 2:** Model parameters

Symbol	Unit	Value	Description
*R*_max_	mg/L	30	Maximum resource density
*δ*	per day	0.01	Resource renewal rate
*Q*	—	1	Maximum ingestion exponent
*P*	—	1	Maintenance exponent
*M*	g/day	0.1	Maximum ingestion constant
*T*	g/day	0.01	Maintenance constant
*μ*_c_	per day	0.0015	Background mortality
*μ*_j_	per day	0.0	Additional juvenile mortality
*μ*_a_	per day	0.0	Additional adult mortality
*σ*	—	0.5	Assimilation efficiency
*H*	mg/L	3	Half‐saturation density
*s*_b_	g	0.1	Body mass at birth
*s*_j_	g	1	Body mass at maturation
*s*_r_	g	1	Reference body mass
*s*_m_	g	10	Maximum body mass

### 2.2 Model analysis

We used PSPManalysis (De Roos, 2018), a software package for the analysis of physiologically structured population models to calculate model equilibria as a function of model parameters. PSPManalysis solves for the resource density ($\tilde{R}$) and population birth rate ($\tilde{b}$) at equilibrium by integrating repeatedly a coupled set of ordinary differential equations that describe the growth, survival, cumulative resource ingestion and cumulative reproduction until the equilibrium condition $R_{0}\left(\tilde{R}\right)\;=\;1$ is satisfied. Here, $R_{0}$ represents the expected lifetime reproductive success of a single individual (see De Roos, 2018, for more details). Equilibrium analysis was complemented with the Escalator Boxcar Train (EBT) method to study transient and non‐equilibrium dynamics (De Roos, 1988). The EBT method calculates population dynamics as a function of time by dividing the size distribution into cohorts of similarly‐sized individuals and for every time step calculating the growth, mortality and reproduction for each cohort, as a function of the body mass of individuals within that cohort and resource density.

To study evolutionary dynamics, we used the framework of adaptive dynamics (Geritz et al., 1998; Metz, 2012; Metz, Geritz, Meszéna, Jacobs, & Van Heerwaarden, 1995), which assumes that mutation limited evolution proceeds according to subsequent trait substitutions in the direction of the selection gradient. This can result in an evolutionary singular strategy (ESS) if the selection gradient becomes zero. PSPManalysis calculates the selection gradient and detects and classifies ESSs according to their stability properties as discussed in Geritz et al. (1998). Since our model has a one‐dimensional environment, all ESSs are convergence and evolutionary stable (CSSs; continuously stable strategies). Furthermore, PSPManalysis is used to calculate evolutionary isoclines, which denote the ESS‐value of one model parameter as a function of a second model parameter.

## 3 RESULTS

### 3.1 Ecological dynamics as function of *Q* and *P*

**Figure 1:**
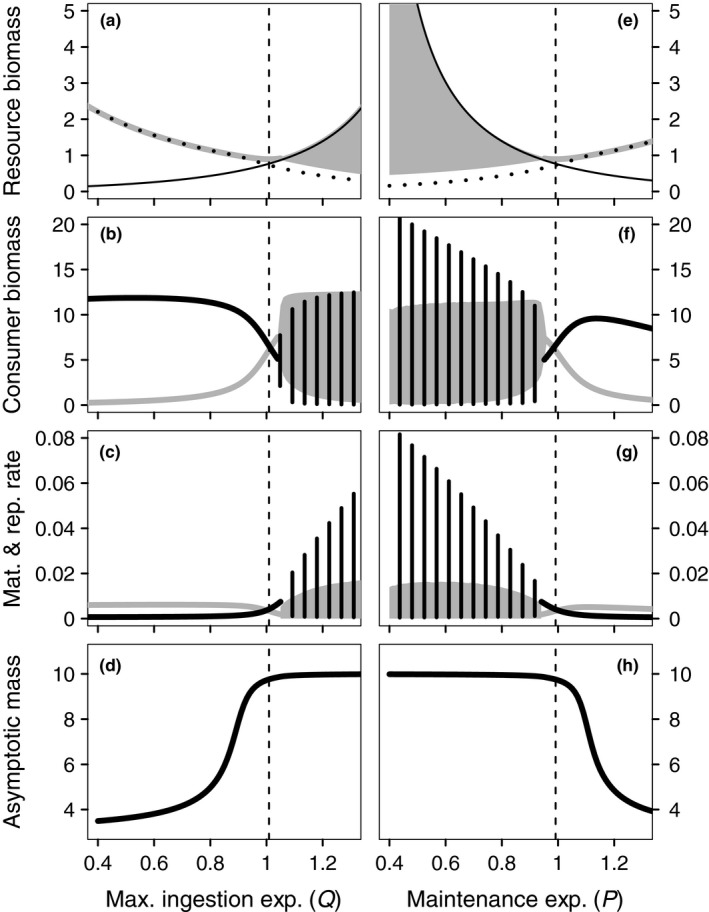
Model dynamics as a function of the maximum ingestion scaling exponent *Q* (left panels with *P* = 1) and the maintenance rate scaling exponent *P* (right panels with *Q* = 1). Thick lines indicate stable model equilibria, while the solid‐filled and dashed areas show the range and extent of population cycles. (a, e) Resource biomass (grey thick lines and shading) and the maintenance resource density for the smallest (solid black lines) and largest individuals (dotted black lines) occurring in the population. (b, f) Adults (black) and juvenile (grey) consumer biomass. (c, g) Total population reproduction (black) and maturation (grey) rates in biomass. (d, h) Body mass of largest individuals in the population (asymptotic body size). The vertical dashed lines show the position of the continuously stable strategies (CSS) of *Q* in panels a:d, and the CSS of *P* in e:h. All other parameters as in Table 2

The model dynamics as a function of *Q* and *P* show a similar pattern. For *P* > *Q*, the maintenance rate increases faster with body mass *s* than the maximum ingestion rate (Q<1 in Figure 1a–d and P>1 in Figure 1e–h), in which case small individuals produce more biomass per unit of existing biomass and require less resources to cover their maintenance requirements than large individuals. A stable equilibrium results in which the asymptotic body mass is determined by the size at which adult individuals spend all assimilated biomass on maintenance. Consequently, when P>Q the resource density at equilibrium coincides with the MRD of the largest individuals in the population (Figure 1a,e), which are well below the maximum possible body mass of $s_{m}\;=\;10$ (Figure 1d,h).

The low mass‐specific biomass production of adults is insufficient to compensate for adult biomass loss through mortality and there is a net biomass loss in the adult stage. This net loss is compensated for by a net biomass gain in the juvenile stage, where the biomass production exceeds the biomass loss through mortality. The discrepancy in biomass production between the two life stages translates to a discrepancy in total biomass reproduction and maturation rates, where maturation exceeds reproduction because the net production of biomass occurs in the juvenile stage (Figure 1c,g). Juveniles therefore grow rapidly but growth and reproduction of adults is slow. As a result, the population is dominated by adult biomass (Figure 1b,f).

If the maximum ingestion rate increases faster with body mass than the maintenance rate (Q>P), larger individuals both have a higher mass‐specific biomass production rate and a lower MRD (Q>1 in Figure 1a–d and P<1 in Figure 1e–h). This induces population cycles driven by adults that are close to the maximum size and hinder growth of newborn individuals (Figure 1d,h). Growth of newborns occurs only when background mortality has diminished adult density to allow the resource to increase above the MRD of newborn individuals. This explains the coincidence of the resource density with the MRD of newborn individuals (thin solid lines in Figure 1a,e). Because ingestion increases faster with size than maintenance, the growing juveniles decrease the resource density and inhibit growth of later cohorts. If abundant enough, these later cohorts will either catch up with the earlier produced individuals when the resource density increases, or die due to background and starvation mortality. Adult‐driven cycles exist for P=1 and Q>1, and for Q=1 and P<1. Their amplitude and period increases with increasing difference between *Q* and *P*.

### 3.2 Evolutionary dynamics converge towards *Q* = *P*

An ESS exists for values of *Q* and *P* where the equilibrium resource density reaches a minimum (dashed vertical lines in Figure 1). These ESSs are convergent and evolutionarily stable endpoints of evolution (continuously stable strategies; CSSs). Both CSSs are within the parameter region of stable ecological dynamics. Convergence stability within the parameter range of population cycles was confirmed by explicitly assessing the fate of mutant phenotypes in the cyclic attractor of the resident. Only mutants with a trait value closer to the CSS were able to invade and replace the resident population. In the following we refer to the CSS of *Q* as $\overset{¯}{Q}$ and to the CSS of *P* as $\overset{¯}{P}$.

**Figure 2:**
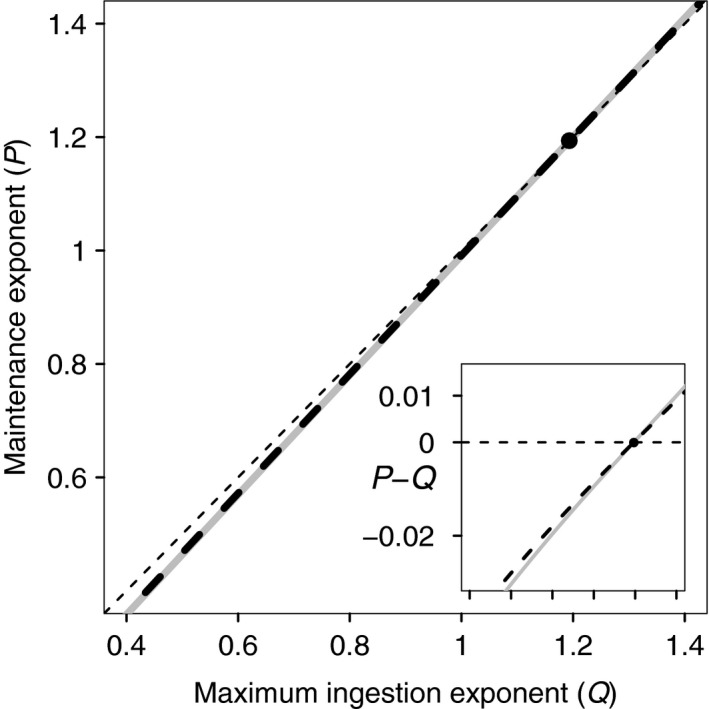
Evolutionary isoclines, showing the value of $\overset{¯}{Q}$ (the continuously stable strategies [CSS] of *Q*) as a function of *P* (grey line), and $\overset{¯}{P}$ (the CSS of *P*) as a function of *Q* (black dashed line) in the *Q* −* P*—plane. Thin dashed lines represent *Q* = *P*. The intersection of both isoclines with the line *Q* = *P* is indicated with a solid point. The inset shows the difference between each of the two evolutionary isoclines and the line *Q* = *P*, as a function of *Q* (horizontal axis range is identical to that of main figure). Isoclines cross exactly when this difference is zero. All other parameters as in Table 2

The value of $\overset{¯}{Q}$ as a function of *P*, as well as the value of $\overset{¯}{P}$ as a function of *Q*, is shown in the Q-P‐parameter space in Figure 2. These evolutionary isoclines appear to be on top of each other, but closer inspection reveals that they cross exactly at $\overset{¯}{Q}\;=\;\overset{¯}{P}$ (inset Figure 2). The small difference between both isoclines means that the CSS‐value of one exponent is approximately equal to the value of the other, non‐evolving, exponent. Therefore, the evolving exponent will approximate the value of the non‐evolving exponent. Because the evolutionary isoclines cross exactly at zero difference the two‐dimensional CSS of *Q* and *P* has the property that $\overset{¯}{Q}\;=\;\overset{¯}{P}$. For the default parameters (Table 2), the common CSS‐value is $\overset{¯}{Q}\;=\;\overset{¯}{P}\approx 1.19$. Because $\overset{¯}{Q}\;=\;\overset{¯}{P}$, the MRD in the CSS does not change with body mass. However, since $\overset{¯}{Q}\;=\;\overset{¯}{P}>1.0$ the mass‐specific biomass production rate increases with body mass. In the Supporting Information Figure S1, we show that the result of $\overset{¯}{Q}\;=\;\overset{¯}{P}$ is independent of the scaling reference size, $s_{r}$, while the joined value of $\overset{¯}{Q}$ and $\overset{¯}{P}$ does depend on $s_{r}$. Increasing $s_{r}$ leads to a decrease of $\overset{¯}{Q}\;=\;\overset{¯}{P}$. This result still holds if we apply separate scaling reference masses for maximum ingestion and maintenance (Supporting Information Figure S1). In addition, we explore the effect on $\overset{¯}{Q}$ and $\overset{¯}{P}$ of parameters $R_{\max}$, δ, *M*,* T*, $\mu_{c}$, *H* and σ in Supporting Information Figure S2. None of these parameters leads to a difference between the evolved scaling exponents. Moreover, the only parameters that slightly affect the values of $\overset{¯}{Q}$ and $\overset{¯}{P}$ are the maintenance constant (*T*) and the background mortality rate ($\mu_{c}$). The effect of the remaining model parameters on $\overset{¯}{Q}$ and $\overset{¯}{P}$ is described below.

### 3.3 Effect of juvenile and adult size ranges

**Figure 3:**
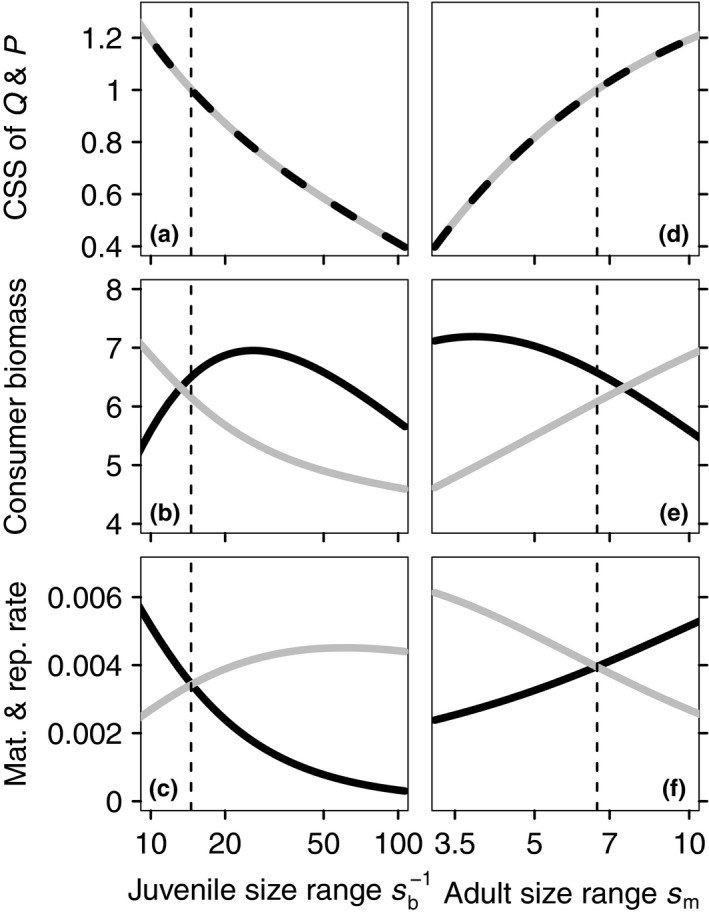
Continuously stable strategies‐values of scaling exponents of maximum ingestion *Q* (grey solid line) and maintenance rate *P* (black dashed line) in panels (a and d), as a function of the juvenile size range, parameterized by $s_{b}^{-1}$ (a–c) and the adult size range, parameterized by $s_{m}$ (d–f). (b, e) Adult (black) and juvenile (grey) consumer biomass. (c, f) Population‐level reproduction (black) and maturation (grey) rates in biomass. The vertical dashed lines indicate the value of $s_{b}^{-1}$ (a–c) and $s_{m}$ (d–f) where *Q* and *P* evolve to 1

The evolutionary convergence of the scaling exponents to a common CSS‐value is robust against changes in size at birth $s_{b}$ and the maximum body size $s_{m}$, although the value of the common CSS‐point is influenced by these size parameters. Figure 3 shows that increasing the size range of a life stage changes $\overset{¯}{Q}$ and $\overset{¯}{P}$ such that the mass‐specific biomass production of this life stage increases. A decrease in $s_{b}$ leads to a larger juvenile size range and triggers an evolutionary response of a decrease in $\overset{¯}{Q}$ and $\overset{¯}{P}$. This increases mass‐specific biomass production for juveniles, increases growth and maturation rates and leads to a lower juvenile biomass density (Figure 3b,c). Alternatively, a larger adult size range (increase in maximum size $s_{m}$) triggers an evolutionary response of increasing values of $\overset{¯}{Q}$ and $\overset{¯}{P}$ (Figure 3d). Consequently, the increased biomass production of adults increases reproduction and leads to lower adult biomass density (Figure 3e,f). For one specific combination of $s_{b}$ and $s_{m}$ does selection on *Q* and *P* lead to $\overset{¯}{Q}\;=\;\overset{¯}{P}\;=\;1$ (dashed vertical lines in Figure 3). Only for this combination of size parameters does the model predict the mass‐specific biomass production to be independent of body size.

### 3.4 Effect of stage‐specific mortality

**Figure 4:**
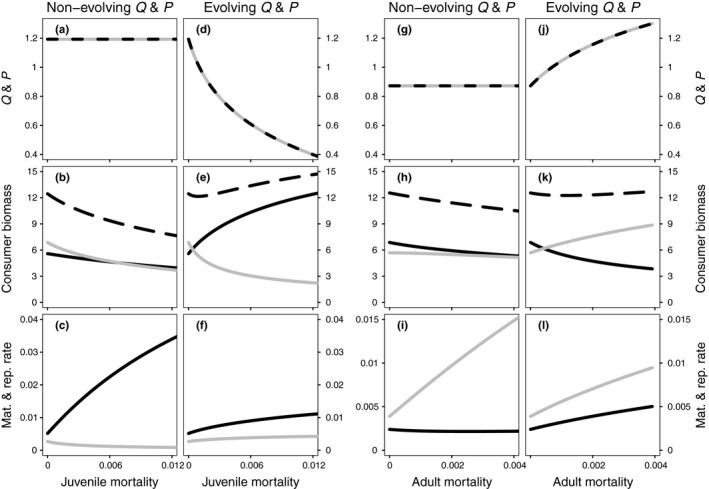
Equilibria as a function of increasing stage‐specific mortality for juveniles ($\mu_{j}$; left six panels) and adults ($\mu_{a}$; right six panels). Each set of six panels compares the population response for non‐evolving scaling exponents (*Q* and *P* fixed at their continuously stable strategies‐values for no additional mortality), with the case in which the scaling exponents respond adaptively to increasing stage‐specific mortality ($\overset{¯}{Q}$ and $\overset{¯}{P}$). Top panels: values of maximum ingestion (*Q*, grey solid line) and maintenance scaling exponent (*P*, black dashed line), middle panels: adult (solid black lines), juvenile (grey lines) and total (dashed black lines) consumer biomass, bottom panels: population‐level rates of reproduction (black lines) and maturation (grey lines) in terms of biomass. Left six panels: default parameters (Table 2) where $\overset{¯}{Q}\;=\;\overset{¯}{P}\approx 1.193$ at $\mu_{j}\;=\;0.0$. Right panels: default parameters (Table 2), in addition to $s_{b}\;=\;0.05$, for which $\overset{¯}{Q}\;=\;\overset{¯}{P}\;=\;0.872$ at $\mu_{a}\;=\;0.0$

The response to increasing stage‐specific mortality is shown in Figure 4. The evolutionary response of $\overset{¯}{Q}$ and $\overset{¯}{P}$ (Figure 4d–f and j–l) is compared with the population response to increasing mortality in case the scaling exponents do not evolve (Figure 4a–c and g–i), but are instead fixed at their CSS‐value for no additional stage‐specific mortality. At $\mu_{j}\;=\;0$, this causes a higher mass‐specific biomass production for adults compared to juveniles and, consequently, the population‐level reproduction rate exceeds the population‐level maturation rate (Figure 4c,f). For additional adult mortality ($\mu_{a}$), the size at birth parameter is set to $s_{b}\;=\;0.05$ and this leads to $\overset{¯}{Q}\;=\;\overset{¯}{P}\approx 0.872$ for $\mu_{a}\;=\;0$ (Figure 4g,j). Consequently, the mass‐specific biomass production is higher for juveniles and this leads to a larger population‐level maturation rate, compared to the population‐level reproduction rate (Figure 4i,l). When scaling exponents do not evolve, additional stage‐specific mortality leads to a decrease in the rate of the most‐limiting life‐history transition (maturation for increasing juvenile mortality [Figure 4c] and reproduction for increasing adult mortality [Figure 4i]) and an increase in the rate of the other, non‐limiting life‐history transition. However, there is no overcompensatory response of (stage‐specific) biomass density with increasing mortality (Figure 4b,h).

For evolving scaling exponents, additional stage‐specific mortality does not change the evolutionary convergence of $\overset{¯}{Q}$ and $\overset{¯}{P}$ towards a common value, but does influence this common CSS‐value. Increasing juvenile mortality decreases $\overset{¯}{Q}\;=\;\overset{¯}{P}$ (Figure 4d). The response of decreasing $\overset{¯}{Q}$ increases energy assimilation for juveniles, but simultaneously juveniles experience higher maintenance costs due to a decrease in $\overset{¯}{P}$. Nonetheless, the net result is an increase in the mass‐specific biomass production rate for juveniles. Accordingly, the population‐level maturation rate increases with increasing juvenile mortality (Figure 4f), which leads to an increase in adult and total consumer biomass (Figure 4e). Instead, the response to increasing adult mortality leads to higher values of $\overset{¯}{Q}$ and $\overset{¯}{P}$ and an increase in juvenile biomass through an increase in the population‐level reproduction rate. Consequently, an overcompensatory response of (stage‐specific) biomass density occurs with increasing mortality when the scaling exponents for maximum ingestion and maintenance respond adaptively to such mortality changes.

## 4 DISCUSSION

Recent studies show considerable variation in the intraspecific scaling of metabolism with body size (Glazier, 2005). Metabolic rate affects competitive ability and therefore changes in competitive ability during ontogeny can arise through changes in the scaling of metabolic rate with body mass. The population and community effects of such size‐dependent changes in competition are well documented (De Roos & Persson, 2013). Here we report the first results on the evolutionary dynamics of the scaling of competitive ability with body size. In case of a trade‐off in the energetics between juvenile and adult individuals, the scaling exponents of maximum ingestion and maintenance evolve to minimize the competitive asymmetry within the population. This is achieved when maintenance and ingestion scale in the same way with body size. Only in this case are all the differently‐sized individuals equal with respect to the resource density they require to cover their maintenance costs (maintenance resource density; MRD). We show that this result is robust against changes in any of the model parameters (Figures 3 and 4, Supporting Information Figures S1 and S2).

We hypothesize that the inability of the current model to produce an evolutionary stable outcome in which the scaling exponents of maintenance and maximum ingestion differ is related to the negative effects of size‐dependent changes in the MRD on the resource use efficiency of a single individual. At population equilibrium, a single individual has to replace itself and this can only happen if the resource density ($\tilde{R}$) exceeds the MRD of all consumer individuals. Consequently, if the MRD is a size‐dependent function of consumer body size, $\tilde{R}$ has to increase beyond the maximum of this function. Let's consider the case that maintenance increases faster with body size compared to maximum ingestion (P>Q). The MRD is an increasing function of body size and $\tilde{R}$ exceeds the MRD of the largest consumer individuals (Figure 1). Compared to the smallest individuals, the largest individuals suffer most from resource limitation, as their biomass production is just above zero. This allows for the invasion of a mutant type with a lower maximum MRD. For this mutant type, the relative increase in biomass production during the phase of strong resource limitation (when large), will outweigh the relative decrease in biomass production when small. Consequently, this mutant will oust the resident, since it is able to replace itself with a lower $\tilde{R}$‐value. Individual resource use efficiency will therefore be most efficient when the MRD is a constant function of body size. Although we did not prove this directly, we suspect that this mechanism is responsible for the strong selection towards equal scaling exponents of maintenance and maximum ingestion.

Obviously, a trade‐off is required to constrain the evolution of the scaling exponents of maintenance and maximum ingestion. By default, we have adopted a juvenile–adult trade‐off by setting the point of rotation of the power‐law functions that describe these scaling exponents to the size at maturation. If we would use, for example, the size at birth instead, an increase in *Q* and a decrease in *P* would lead to, respectively, an increase in maximum ingestion and a decrease in the maintenance rate for all consumer individuals. This would inevitably cause runaway selection towards higher *Q* and lower *P*, which is clearly unrealistic. Although there is no good empirical justification for a juvenile–adult trade‐off, it does allow us to study the selection pressures that arise from unequal scaling exponents of maintenance and maximum ingestion.

The evolutionary prediction of equal scaling exponents of maintenance and maximum ingestion is at odds with the existing theories about ontogenetic growth. These theories assume an isometric increase of maintenance costs (P=1) and a sublinear allometry of maximum ingestion rates (Q=3/4 or Q=2/3 Kooijman, 2010; Van der Meer, 2006; West et al., 2001). Such a combination of scaling exponents leads to a decrease in the MRD with individual body size. We show that this leads to negative selection on the maintenance exponent and positive selection on the maximum ingestion exponent. Also, our results show that when either *Q* or *P* is fixed, the other exponent still evolves to a value close to the evolutionarily constrained scaling exponent. Therefore, in this model there can only be a difference between both exponents when they are both constrained and non‐evolvable. Selective change in one or both exponents will eventually bring them together.

Despite the difficulties of measuring maintenance and ingestion rates, most data suggest that maintenance rates indeed scale faster with body size than ingestion rates, although considerable variation exists (Glazier, 2005; Maino & Kearney, 2015). One difficulty in measuring maintenance metabolic rate is that resting individuals can still invest in growth or reproduction. Therefore, measures like resting or basal metabolic rate still include overheads costs for these investments (Kooijman, 1986; McCauley, Murdoch, Nisbet, & Gurney, 1990). Even if maintenance rate would be measured reliably, the resulting maintenance rate exponent often shows considerable variation (Glazier, 2005). Likewise, scaling of ingestion also varies between groups. The two‐thirds scaling predicted by DEB theory appears to hold for non‐volant mammals, but the pooled mass exponent for both non‐volant mammals and birds is 0.73 and for birds alone it is even higher (Kearney & White, 2012). Significant variation in the scaling of ingestion was also observed for insects (Maino & Kearney, 2015). Due to this variation, a comparison between the two can only be informative when measurements are performed under identical conditions and for the same species or even the same population. Such a comparison exists for *Daphnia* sp., in which maintenance rates increase superlinear with body mass (>1) due to contributions to carapace formation and ingestion follows an overall exponent of 0.73 (Gurney, McCauley, Nisbet, & Murdoch, 1990; McCauley et al., 1990).

Also ontogenetic growth data suggest that the maintenance rate scaling exceeds the ingestion rate scaling, as this leads to the often observed pattern of decreasing ontogenetic growth rates with increasing size (Peters, 1983; Ricklefs, 2003). Equal scaling exponents would lead to an exponential growth pattern, which are less often observed (Kooijman, 2010). However, a decreasing ontogenetic growth rate with body size can also arise from an increasing energy allocation towards reproduction (Barneche, Robertson, White, & Marshall, 2018) and does not necessarily imply a lower ingestion rate scaling. Another way to assess the scalings of maintenance and ingestion rates is by using the scaling of the MRD with body size. Several empirical studies indicate that the MRD is an increasing function of body size, which implies a steeper scaling of maintenance compared to ingestion and leads to a competitive advantage of small individuals (Aljetlawi & Leonardsson, 2002; Byström & Andersson, 2005; Hjelm & Persson, 2001). In conclusion, both data and theoretical models on ontogenetic growth indicate a steeper body‐size scaling of maintenance compared to ingestion and this contradicts the evolutionary prediction of equal scaling exponents.

One simple explanation for this discrepancy is that the scaling exponents are constrained and therefore cannot evolve, but there is at least some evidence against this explanation. First of all, the range of observed intraspecific scaling exponents does suggest there is variation for selection to act on (Glazier, 2005). This variation has been related to lifestyle, activity, growth form, temperature and predation (Glazier, 2005, 2006; Glazier et al., 2015; Hirst et al., 2014; Killen et al., 2010). For example, body shape changes can influence the intraspecific scaling of metabolic rate (Okie, 2013). As shown by Hirst et al. (2014) growth in three dimensions (isomorphic growth) is related to low scaling exponents, while one‐dimensional growth (elongation) relates to exponents around one. These findings favour theories that assume that metabolic scaling is determined by transport of materials across surface membranes and indicate that changes in growth form can influence the scaling of resource supply rates. Selection would hence be able to change the scaling of maximum ingestion with body size by altering the dimension of ontogenetic growth.

Secondly, Glazier et al. (2011) illustrate that adaptations to different environments can lead to different scaling exponents. These authors show how the scaling of resting metabolic rate in the amphipod *Gammarus minus* depends on the presence of fish predators. Individuals from three populations that naturally co‐occur with a predatory fish have a lower scaling exponent than individuals from two populations in which these predators are absent. The lower scaling exponents resulted in a higher metabolic rate for small individuals and a lower metabolic rate for large individuals. This led to faster growth to lower asymptotic sizes of individuals in fish‐exposed populations (Glazier et al., 2011). These results correspond to our evolutionary predictions in case of increasing juvenile mortality, which lowers the evolutionary equilibrium of the scaling exponents of maintenance and maximum ingestion and leads to a higher growth potential of juveniles at the expense of adult growth potential.

The inability of the current model to reproduce evolutionary stable scaling exponents that match empirical observations suggests that our model misses a crucial aspect of biological reality. Indeed, we have used a basic size‐structured consumer–resource model, with a single type of resource and a simple DEB model to describe consumer energetics. Although similar DEB models are able to reproduce empirical patterns of ontogenetic growth, reproduction and metabolism from the principles of energy and mass conservation (Kooijman, 2010), our model appears less successful for explaining the evolution of metabolism and life histories. Likely, this requires incorporating additional ecological complexity besides the elementary consumer–resource interaction studied here. For example, additional ecological interactions might change the evolutionary predictions. In many empirical systems, ontogenetic diet shifts, prey/predator size ratios and interference competition reduce the size dependency of the maintenance resource density, and in this way counteract the negative competitive effect of small individuals on large conspecifics (Aljetlawi & Leonardsson, 2002; Byström & Andersson, 2005; Hjelm & Persson, 2001). Similarly, cannibalism or additional resources types might stabilize size‐dependent changes in the maintenance resource density, by reducing the strength of intraspecific competition. Future research should point out whether additional ecological complexity can allow for an evolutionary stable outcome in which the values of the scaling exponents differ.

Other types of structured population models have proven successful in providing evolutionary predictions of observed life‐history strategies. For example, evolutionary predictions from integral projection models (IPMs) closely match observed flowering decisions of monocarpic perennial plants (Childs, Rees, Rose, Grubb, & Ellner, 2004; Metcalf, Rose, & Rees, 2003; Metcalf et al., 2008). These models differ from our approach, as they are parameterized with functions fitted on growth, reproduction and mortality data (Metcalf et al., 2003) and are not based on energy budget dynamics. The work on monocarpic perennials has revealed which model components are required to successfully predict evolutionary stable strategies of natural populations. One such component is variation in body‐size growth (Metcalf et al., 2003), which is absent in the framework we use. These and other insights from demographic evolutionary models could be used to improve evolutionary predictions of structured population models that are based on explicit energy dynamics.

In a recent study, Barneche et al. (2018) found that in marine fishes reproduction scales with body size with a power larger than 1 (hyperallometrically). In our model, the scaling of reproductive output with body size depends on (a) the evolved scaling exponents of maintenance and maximum ingestion that govern the scaling of biomass production (b) the sigmoidal κ(s)‐function that models allocation of biomass production to growth vs. reproduction. As we show in Supporting Information Figure S3 these two components result in a scaling of realized reproduction rate with body size that closely resembles a hyperallometric scaling, as was found by Barneche et al. (2018).

**Figure 5:**
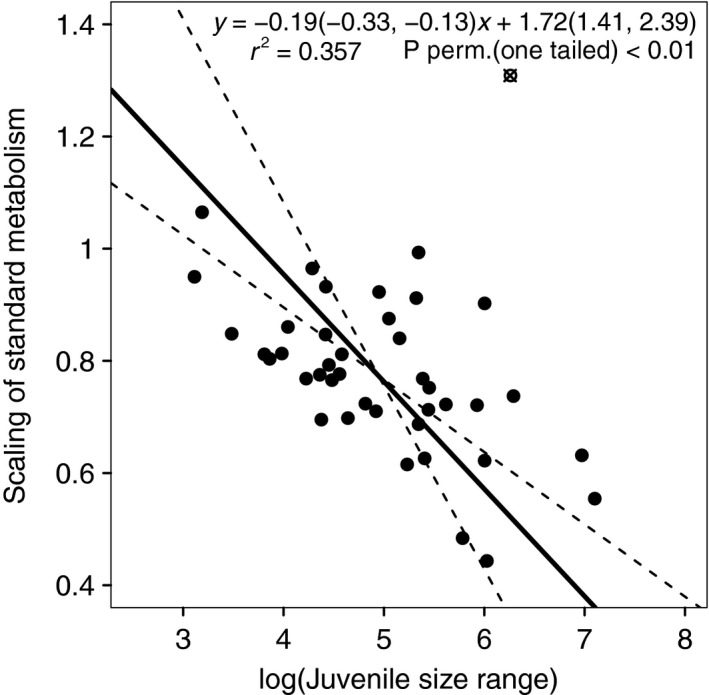
The scaling exponent of standard or routine metabolism with body size of 41 teleost fish species plotted against the logarithm of the ratio of length at maturation and egg diameter, as a measure for the juvenile size range. Data on scaling exponents are from Killen et al. (2010). The lines show a RMA regression with 95% confidence intervals. The point indicated by the open circle with the cross is the eel *Anguilla anguilla* and was not part of the regression equation. See Supporting Information Appendix S2 for a detailed description of data collection and analysis. Data are deposited in Dryad Digital Repository (Hin & De Roos, 2018a)

As a second result, we show how the common CSS‐value of the scaling exponents depends on mortality rates and size ranges of juvenile and adult life stages (Figures 3 and 4). Based on this, we predict that metabolic scaling exponents decrease with the size range and mortality rate of juvenile life stage, while they increase with the size range and mortality rate of the adult life stage. We tested this prediction by using data on scaling exponents of standard or routine metabolic rate for teleost fish as published by Clarke and Johnston (1999) and Killen et al. (2010) and combining these with estimates of length at maturation ($l_{\mathrm{mat}}$) and egg diameter ($l_{\mathrm{egg}}$; as length estimates are most readily available for fish). In total we obtained length estimates for 41 of the 89 species in the original dataset of Killen et al. (2010). In Figure 5 the temperature‐corrected scaling exponents of metabolism are plotted against the logarithms of $l_{\mathrm{mat}}/l_{\mathrm{egg}}$. In agreement with our evolutionary prediction, the scaling exponent of standard metabolic rate decreases significantly with an increase in the juvenile size range, despite the small sample size and the considerable variation that is normally associated with metabolic scaling exponents, egg diameters and maturation sizes (Bagenal, 1971; Kamler, 2005; Killen et al., 2010).

In this study, we ignore the proximate causes that lead to the allometric scaling of maintenance metabolism and ingestion and instead focus on the ultimate, evolutionary causes that are shaped by how individuals interact with each other through their interaction with a shared environment. Such interactions ultimately determine fitness and drive evolutionary change. Although this model describes a basic consumer–resource interaction, it provides a powerful and robust null model against which to evaluate evolutionary considerations regarding the intraspecific scaling of ingestion and maintenance with body size. While the model provides an explanation for the observed variation in the scaling of basal metabolism with body size (Figure 5), the predicted evolutionary convergence of ingestion and maintenance exponents contrast with a substantial amount of empirical findings. Future studies should focus on how energetic models, such as the one described here, can be extended with more ecological realism such that their evolutionary predictions are better in line with real‐world observations.

## AUTHOR'S CONTRIBUTIONS

V.H. and A.M.d.R. designed the research and contributed to later versions of the manuscript. V.H. analysed the model and wrote first version of manuscript. All authors gave final approval for publication.

## Data Availability

Data in Figure 5 are deposited in the Dryad Digital Repository https://doi.org/10.5061/dryad.2fc6677 (Hin & De Roos, 2018a). Code to run the model is available via Zenodo: https://doi.org/10.5281/zenodo.1494830 (Hin & De Roos, 2018b).
