# Supplementary material for: Evolution of size‐dependent intraspecific competition predicts body size scaling of metabolic rate
Source: Funct Ecol. 2018 Dec 20;33(3):479–90. doi: 10.1111/1365-2435.13253 (PMC6472492; doi:10.1111/1365-2435.13253)
Supplement: Supplementary file 1 [file FEC-33-479-s001.pdf]

Functional Ecology

BRITISH ECOLOGICAL SOCIETY

Can variation in the body-size scaling of metabolic rate be explained through consumer-resource interactions?

Vincent Hin & André M. de Roos

All organisms need to spend a certain amount of their energy on self-maintenance. The rate at which they do so is called the maintenance rate, or basal metabolic rate. Body size is the main determinant of the maintenance rate, although considerable variation exists in how the maintenance rate changes with body size when organisms grow.

Animals acquire energy through ingestion and assimilation of food and this rate is also mainly determined by body size. The amount of energy left-over from ingested food, after subtraction of maintenance costs, can be used for body-size growth or reproduction. In many species, the maintenance rate increases more rapidly than the ingestion rate when the individuals grow in size. Therefore, larger individuals have proportionally less energy available to invest into growth or reproduction and also require more food to cover their maintenance costs.

When differently-sized individuals of the same species compete for the same resource, the smaller individuals can reduce resources to a level that is insufficient to cover the maintenance costs of the larger individuals. This leads to starvation of large individuals and can induce recurrent large-scale fluctuations in population density.

By modelling the interaction between differently-sized individuals and their shared food source, we show that a difference between the scaling of maintenance and ingestion with body size actually represents an evolutionary unstable state. If selection operates on either the ingestion scaling, the maintenance scaling, or both, this will ultimately lead to an identical scaling of maintenance and ingestion with body size. Such a pattern is not in accordance with empirical observations and we discuss how additional ecological interactions besides resource competition might account for this discrepancy.

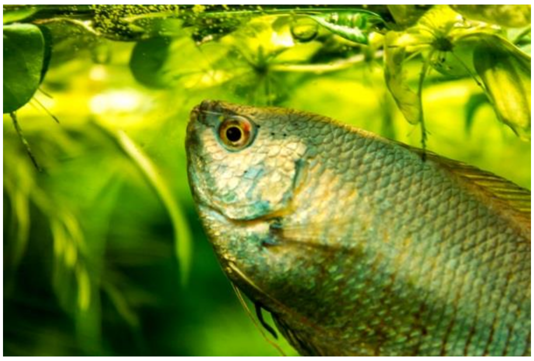

Fish under water. By Michael Palmer [CC BY-SA 4.0 from Wikimedia Commons.

As a second result, the evolved common scaling of ingestion and maintenance will vary depending on how mortality rates change with body size and on the extent of body-size growth early versus late in life. More specifically, the model predicts that substantial body size growth before maturation will lead to a lower scaling of maintenance and ingestion with body size. This prediction is supported by data on the scaling of basal metabolic rate of bony fish.
