# Supplementary material for: Evolution of size‐dependent intraspecific competition predicts body size scaling of metabolic rate
Source: Funct Ecol. 2018 Dec 20;33(3):479–90. doi: 10.1111/1365-2435.13253 (PMC6472492; doi:10.1111/1365-2435.13253)
Supplement: Supplementary file 2 [file FEC-33-479-s002.pdf]

SUPPORTING INFORMATION

of

Evolution of Size-Dependent Intraspecific Competition Predicts Body Size Scaling of Metabolic Rate

Vincent Hin[1,*] & André M. de Roos[1]

1. Institute for Biodiversity and Ecosystem Dynamics, University of Amsterdam, P.O. Box 94248, 1090 GE Amsterdam, The Netherlands

* Corresponding author; e-mail: V.Hin@uva.nl.

Appendix S1: Model Parameters

Body size of the consumer is expressed in grams (g) and biomass densities are expressed in milligrams per liter (mg L^{-1}). Across differently-sized species, mass-specific maximum ingestion, mass-specific maintenance and mortality rates are inversely proportional to the quarter power of adult body size (Kleiber, 1932; Peters, 1983; Savage et al., 2004). Taking time in days and adult body weight in grams, representative proportionality constants of these scaling relationships for invertebrate species are 0.1, 0.01 and 0.0015 for maximum ingestion, maintenance and mortality, respectively (De Roos & Persson, 2013). For the parameterization we take the characteristic adult body mass to equal the size at maturation, for which we adopt a value of 1 gram. Hence $M = 0.1$, $T = 0.01$ and $\mu_c = 0.0015$. A value of 0.01 is adopted for the resource renewal rate δ, such that resource turn-over rate equals the mass-specific maintenance rate of an individual with size s_j. Only a change in the ratios between these four rates changes model predictions, as changing their absolute values all with the same factor only scales the unit of time. Values for the volume related parameters are $R_{max} = 30$ mg L^{-1} and $H = 3$ mg L^{-1} (Table 2, main text). The value for H is derived from zooplankton grazing rates as presented by Hansen et al. (1997) and R_{max} is assumed one order of magnitude larger than H (De Roos & Persson, 2013). As long as the ratio of these volume-related parameters remains constant a change in these parameters is equivalent to a scaling of the volume in which the consumer-resource interaction takes place. This does not qualitatively change model predictions. Default parameters for size at birth and maximum size are 0.1 and 10, but these values are changed during model analysis. The scaling exponents Q and P are varied throughout the analysis, but their values are limited to the range 0.4–1.3, which is the observed range of intraspecific scaling exponents found by Clarke & Johnston (1999) for post-larval teleost fish.

Figure S1

We assume a juvenile-adult trade-off in both maximum ingestion rate and maintenance rate. This trade-off is implemented by parameterizing the power functions that describe these rates at the size at maturation. When another reference size than the size at maturation is used to fix these power functions, both exponents still evolve towards a common value. This CSS-value increases with decreasing reference size, see Figure S1 (left panel).

Moreover, using different scaling reference sizes for maintenance and maximum ingestion does not influence this result. Denoting the scaling reference mass for maximum ingestion by s_{r_I}, the resource ingestion function becomes:

$$I(R, s) = M\left(\frac{s}{s_{r_I}}\right)^Q \frac{R}{R+H}$$

Similarly denoting the scaling reference mass for maintenance by s_{r_M}, the biomass production function becomes:

$$\Omega(R, s) = \sigma I(R, s) - T\left(\frac{s}{s_{r_M}}\right)^P$$

Figure S1 shows how the CSS-values of Q and P change when only changing s_{r_I} (middle panel) or s_{r_M} (right panel). Although the CSS-values respond rapidly to changes in s_{r_I} and s_{r_M}, the evolved scaling exponents remain approximately identical to one another.

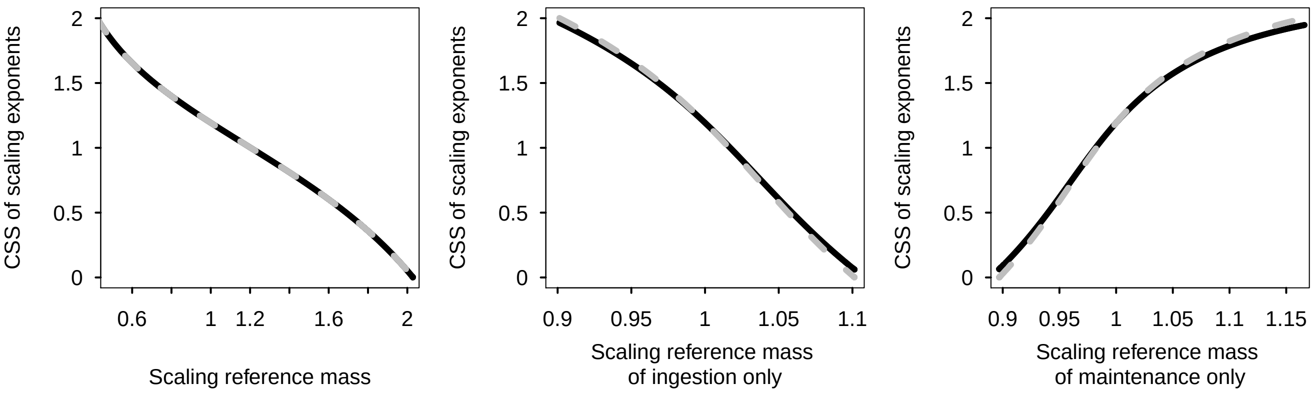

Figure S1: CSS-values of the scaling exponents of maximum ingestion Q and maintenance P, as a function of the scaling reference mass s_r (left), the separate scaling reference mass for maximum ingestion s_{r_I} (with $s_{r_M} = 1$), or the separate scaling reference mass for maintenance s_{r_M} (with $s_{r_I} = 1$). All other parameters as in Table 2 (main text)

Figure S2

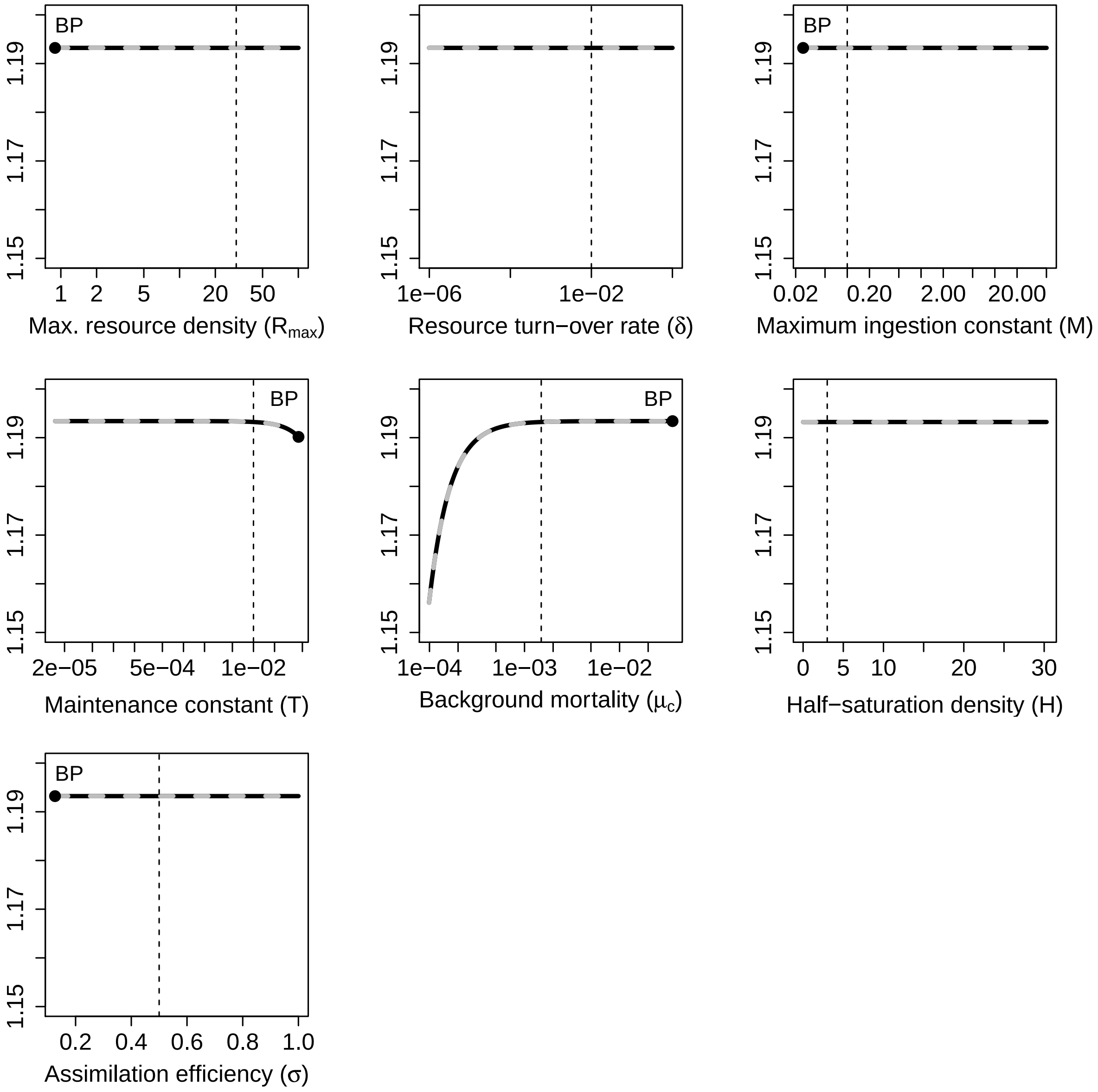

Figure S2: The CSS-values of the maintenance (P) and maximum ingestion (Q) scaling exponents as a function of seven model parameters, as indicated on each horizontal axis. The main result that these scaling exponents evolve towards the same value is independent of changes in any of these parameters. Moreover, changes in five out of seven of these parameters do not influence the CSS-values. Only the maintenance constant T and the size-independent mortality rate μ_c have a slight impact on the CSS-values of Q (solid gray lines) and P (black dashed lines). Vertical dashed lines indicate default parameter values (main text Table 2)

37 # Figure S3

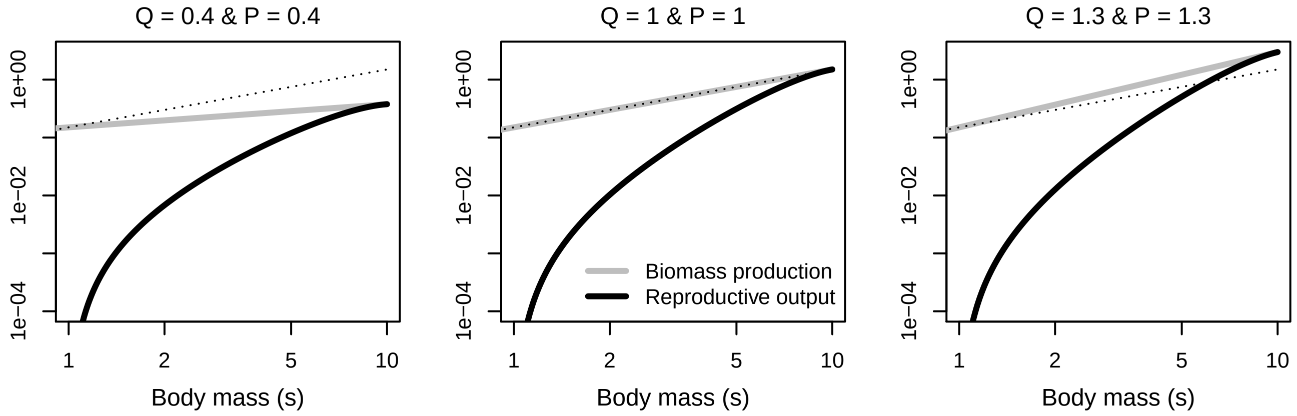

Figure S3: The scaling of the biomass production rate $\Omega^{+}(R, s)$ and the reproductive output in terms of biomass: $(1-\kappa(s))\Omega^{+}(R, s)$, as a function of the adult body-mass range s_j to s_m, plotted on a log-log scale. Reproductive output scales more steeply with body mass compared to the biomass production rate, due to the increasing allocation towards reproduction with increasing size. For the range of scaling exponents considered (0.4 – 1.3), reproductive output scales approximately hyperallometrically with body mass. The thin dashed line shows an isometric scaling of biomass production: $\Omega^{+}(R, s)$ with $Q = P = 1$. All other parameters at default values (main text Table 2) and $R = 3$.

Appendix S2: Data Collection and Analysis

Data collection

Data on the relationship between metabolic rate and body mass were taken from the supporting information of Killen, Atkinson, & Glazier (2010). This dataset provides estimates on metabolic scaling exponents (b) and metabolic activity level (L), defined as "the mass-specific metabolic rate estimated at the body mass corresponding to the midpoint of the allometric relationship" (Killen et al., 2010) for 89 species of Teleost fish. These data are an extension of the data provided by Clarke & Johnston (1999) and selection criteria handled by both Killen et al. (2010) and Clarke & Johnston (1999) were aimed at selecting rates of standard or routine metabolism. Only data on resting, post-larval, fasted animals measured in absence of additional stressors and after a 24h acclimation period were accepted (Killen et al., 2010). Additionally, the temperature (T) at which metabolism was measured should be within the natural temperature range of the species and the metabolism should be measured over a suitable body-mass range (see Clarke & Johnston (1999) and Killen et al. (2010) for a more detailed description of acceptance criteria).

To test the hypothesis that the scaling of metabolic rate changes with the ratio between size at birth and size at maturation we collected data on egg diameter (l_{egg}) and length at maturation (l_{mat}) for the species in the dataset of Killen et al. (2010). Length instead of mass estimates were used since the former are more readily available for fish. Furthermore, any pattern in the length ratios will remain intact after a non-linear transformation to mass ratios. Data collection was carried out at the species level and the estimates for l_{mat} and l_{egg} are generally not from the same source, nor is any of the two directly derived from populations or individuals that were used for the metabolic rates measurements. There is considerable variation in both size at maturation and egg size between individuals of the same population, between different populations of the same species and with size and age of the parents (Bagenal, 1971; Bøhn, Sandlund, Amundsen, & Primicerio, 2004; Bonislawska, Formicki, Korzelecka-Orkisz, & Winnicki, 2001; Kamler, 2005; Wallace & Aasjord, 1984). This variation could not be controlled for and is taken for granted since estimates on our variables of interest (l_{egg}, l_{mat} and b) are not available for the same individuals.

Various sources report different estimates of l_{egg} and l_{mat} for the same species and these data were averaged to arrive at a species-specific prediction. Main sources for size at maturation estimates were Winemiller & Rose (1992), King & McFarlane (2003) and Fishbase (Froese & Pauly, 2016). Fishbase estimates were collected using R (R Core Team, 2015) with the package 'rfishbase' (Boettiger, Cham-

berlain, Temple Lang, & Wainwright, 2016; Boettiger, Lang, & Wainwright, 2012). Single estimates for l_{mat} were preferred over range estimates and the latter were only used when both minimum and maximum values were reported. Estimates of l_{egg} were mainly derived from Winemiller & Rose (1992), King & McFarlane (2003) and Russel (1976). Additional searches for egg size were performed for species if size at maturation estimates were provided in the sources mentioned above. This resulted in a total of 41 species with estimates of l_{egg}, l_{mat} and b. All data and data source are deposited in Dryad Digital Repository (Hin & De Roos, 2018).

Data analysis

Killen et al. (2010) show an effect of the temperature (T) at which the metabolic measurements where performed on both the scaling of metabolism with body size (b) and on the metabolic level (L). Furthermore, the authors show an effect of $\log(L)$ on b. To correct for these dependencies in our analysis we calculate the effect of T on the $\log(L)$ for the original 89 species described in Killen et al. (2010). This analysis is identical to the one described in the Figure S1 of the supporting information of Killen et al. (2010) and gives an ordinary least squares (OLS) regression of $\log(L) = 0.0653T + 3.17$ (slope $P < 0.0001$ and $R^2 = 0.510$). We used the residuals of the regression of $\log(L)$ against T relative to the prediction for $\log(L)$ at $T = 15°C$ to calculate temperature-corrected estimates of $\log(L)$. These temperature-corrected values are referred to as $\log(L_{Tcorr})$. We related $\log(L_{Tcorr})$ to the scaling exponents of metabolic rate of the original dataset of Killen et al. (2010). Contrary to the values of L the temperature-corrected values do not show a clear relationship with b (slope $= -0.0300$, $P = 0.176$, $R^2 = 0.0212$), indicating that the original effect of $\log(L)$ on b as reported by Killen et al. (2010) was mediated by temperature and not through an effect of $\log(L)$ directly. Due to the lack of a clear correspondence between the temperature-corrected values of $\log(L)$ on b we refrained from correcting estimates of b to the metabolic level L. Instead, we only controlled for a direct effect of T on b. This was done by recalculating the OLS regression between b and T as reported in Figure S1b of Killen et al. (2010) and using the residuals of this regression equation ($b = -0.005872T + 0.875878$, slope $P < 0.0001$, $R^2 = 0.198$) relative to the predicted scaling exponent at $15°C$. These temperature-corrected values were calculated with the full dataset as reported by Killen et al. (2010) and used to test the hypothesis that the scaling of maintenance metabolism is negatively related with the ratio between size at maturation and size at birth for species for which these latter estimates were available.

We used ranged major axis (RMA) regression to calculate the correlation between $\log(l_{mat}/l_{egg})$ and the temperature-corrected scaling exponent b_{Tcorr} (main text Figure 5). Since RMA regression is

sensitive to the presence of outliders (Legendre, 2014), Cook's distance was calculated from an OLS regression of b_{Tcorr} on $\log(l_{mat}/l_{egg})$. The estimates for the eel (*Anguilla anguilla*) had a Cook's distance of 0.697, which was 6.2 times the value of the second highest Cook's distance. Therefore, we decided to exclude the eel from further analysis, since it has a disproportionally large effect on the estimates of the OLS regression. The RMA regression line relating the temperature-corrected scaling exponent of metabolism to the juvenile size-range is: $b_{Tcorr} = -0.19 \log(l_{mat}/l_{egg}) + 1.72$, with $n = 40$, $R^2 = 0.357$ and a one-tailed permutation test of the slope of $P < 0.01$ (main text Figure 5). This negative correlation supports the evolutionary prediction that an increased juvenile size-range through either a smaller size at birth (measured as egg diameter) or a larger size at maturation should result in a lower scaling exponent of metabolism with body size.

References

Bagenal, T. B. (1971). The interrelation of the size of fish eggs, the date of spawning and the production cycle. *Journal of Fish Biology*, *3*, 207–219, https://doi.org/10.1111/j.1095-8649.1971.tb03665.x

Boettiger, C., Chamberlain, S., Temple Lang, D., & Wainwright, P. (2016). *rfishbase: R Interface to 'FishBase'. R package version 2.1.0.1. https://github.com/ropensci/rfishbase*

Boettiger, C., Lang, D. T., & Wainwright, P. C. (2012). rfishbase: Exploring, manipulating and visualizing FishBase data from R. *Journal of Fish Biology*, *81*, 2030–2039, https://doi.org/10.1111/j.1095-8649.2012.03464.x

Bøhn, T., Sandlund, O. T., Amundsen, P.-A., & Primicerio, R. (2004). Rapidly changing life history during invasion. *Oikos*, *106*, 138–150, https://doi.org/10.1111/j.0030-1299.2004.13022.x

Bonislawska, M., Formicki, K., Korzelecka-Orkisz, A., & Winnicki, A. (2001). Fish egg size variability: biological significance. *Electronic Journal of Polish Agricultural Universities*, *4*

Clarke, A., & Johnston, N. M. (1999). Scaling of metabolic rate with body mass and temperature in teleost fish. *Journal of Animal Ecology*, *68*, 893–905, https://doi.org/10.1046/j.1365-2656.1999.00337.x

De Roos, A. M., & Persson, L. (2013). *Population and Community Ecology of Ontogenetic Development*. Princeton: Princeton University Press, https://doi.org/10.1515/9781400845613

Froese, R., & Pauly, D. (2016). *Fishbase. World Wide Web electronic publication. www.fishbase.org, version (01/2016)*

Hansen, P. J., Bjø rnsen, P. K., & Hansen, B. W. (1997). Zooplankton grazing and growth : Scaling within the 2-2,000- body size range. *Limnol Oceanogr*, *42*, 687–704, https://doi.org/10.4319/lo.1997.42.4.0687

Hin, V., & De Roos, A. M. (2018). Data from: Evolution of size-dependent intraspecific competition predicts body size scaling of metabolic rate. *Dryad Digital Repository*, https://doi.org/doi:10.5061/dryad.2fc6677

Kamler, E. (2005). Parent-egg-progeny relationships in teleost fishes: An energetics perspective. *Reviews in Fish Biology and Fisheries*, *15*, 399–421, https://doi.org/10.1007/s11160-006-0002-y

Killen, S. S., Atkinson, D., & Glazier, D. S. (2010). The intraspecific scaling of metabolic rate with body mass in fishes depends on lifestyle and temperature. *Ecology letters*, *13*, 184–93, https://doi.org/10.1111/j.1461-0248.2009.01415.x

King, J. R., & McFarlane, G. A. (2003). Marine fish life history strategies: applications to fishery management. *Fisheries Management & Ecology*, *10*, 249–264, https://doi.org/10.1046/j.1365-2400.2003.00359.x

Kleiber, M. (1932). Body Size and Metabolism. *Hilgardia*, *6*, 315–353

Legendre, P. (2014). *lmodel2: Model II Regression.* R package version 1.7-2. http://CRAN.R-project.org/package=lmodel2

Peters, R. H. (1983). *The Ecological Implications of Body Size*. Cambridge: Cambridge University Press, https://doi.org/10.1017/CBO9780511608551

R Core Team (2015). *R: A Language and Environment for Statistical Computing*. R Foundation for Statistical Computing, Vienna, Austria

Russel, F. S. (1976). *The Eggs and Plankton Stages of British Marine Fishes*. Oval Road, London: Academic Press

Savage, V., Gillooly, J., Woodruff, W. H., West, G. B., Allen, A. P., Enquist, B. J., & Brown, J. H. (2004). The predominance of quarter-power scaling in biology. *Functional Ecology*, *18*, 257–282, https://doi.org/10.1111/j.0269-8463.2004.00856.x

Wallace, J. C., & Aasjord, D. (1984). An investigation of the consequences of egg size for the culture of Arctic charr, Salvetinus alpinus (L.). *Journal of Fish Biology*, *24*, 427–435, https://doi.org/10.1111/j.1095-8649.1984.tb04813.x

Winemiller, K. O., & Rose, K. A. (1992). Patterns of Life-History Diversification in North American Fishes: Implications for Population Regulation. *Canadian Journal of Fisheries and Aquatic Sciences*, *49*, 2196–2218, https://doi.org/10.1139/f92-242
